# Both, Limited and Often Fatal Systemic Infections Caused by *Leuconostoc* spp. in Older, Previously Ill Men Are Usually Acquired in the Outpatient Setting

**DOI:** 10.3390/microorganisms13071626

**Published:** 2025-07-10

**Authors:** Johanna Butt, Cristian Arva, Stefan Borgmann

**Affiliations:** 1 Department of Internal Medicine II, Ingolstadt Hospital, 85049 Ingolstadt, Germany; johanna.butt@klinikum-ingolstadt.de (J.B.); cristian.arva@klinikum-ingolstadt.de (C.A.); 2 Department of Infectious Diseases and Infection Control, Ingolstadt Hospital, 85049 Ingolstadt, Germany

**Keywords:** anorexia nervosa, albumin, inflammation markers, *Leuconostoc lactis*, *Leuconostoc mesenteroides*, probiotic, starter culture, food production, review article

## Abstract

*Leuconostoc* spp. are vancomycin-resistant Gram-positive cocci that are used in food production and as pre- and probiotics. However, *Leuconostoc* spp. can also cause infections. In the present study, the records of patients with *Leuconostoc* spp. detection between January 2012 and March 2025 were analyzed, inclusive of the underlying risk factors. *Leuconostoc* spp. was isolated from 32 patients (21 male, 11 females), including nine patients with blood culture evidence. In the majority of patients, *Leuconostoc* spp. were obtained on the day of admission to the hospital or in the first few days thereafter, arguing against nosocomial acquisition. The median age of men and women (65.3 and 67.8 years) was similar, but seven of the 14 male patients over the age of 65 had the bacteria in blood culture. The female patients with blood culture evidence had suffered from peripartum thrombophlebitis and from anorexia nervosa (BMI 8.8 kg/m^2^). In contrast, men with *Leuconostoc* spp. in the blood culture had severe, limiting underlying diseases. While the two women survived, five of the seven blood-culture-positive men died. Overall, our results show that *Leuconostoc* spp. is mainly acquired in outpatient settings, but men are at a higher risk of acquisition. Colonized men over the age of 60 with severe underlying diseases have a high risk of systemic infection with a fatal outcome.

## 1. Introduction

Bacteria of the genus *Leuconostoc* are anaerobes but aero-tolerant Gram-positive cocci that appear microscopically as lenticular cocci or coccoid rods in pairs or chains. Morphologically, colonies resemble streptococci or enterococci, do not produce catalase, and often exhibit an alpha hemolysis zone. *Leuconostoc* spp. can also cross-react with antiserum against group D streptococci. As the bacteria are sometimes also able to cleave esculin in the presence of bile or bile salts (bile esculin test positive), *Leuconostoc* spp. are occasionally misinterpreted as enterococci or viridians streptococci. *Leuconostoc* bacteria grow best in the temperature range of 20–30 °C, although growth is also possible at lower temperatures of up to +5 °C [1].

The genus *Leuconostoc* belongs to the family Leuconostaceae, which in turn is assigned to the order Lactobacillales and the class Bacilli and then to the phylum Bacillota [2]. According to the “List of Prokaryotic names with Standing in Nomenclature” [3], the genus *Leuconostoc* currently comprises 31 species and eight subspecies (Figure S1).

Bacteria of the order Lactobacillaes are aerotolerant anaerobes and exhibit a low GC content in the DNA. They also share resistance to glycopeptides, caused by the incorporation of a D-alanyl-D-lactate (D-Ala-D-Lac) depsipeptide instead of the D-Ala-D-Ala dipeptide otherwise incorporated into the cell wall during cross-linking sheets of the murein sacculus [4,5].

Many species of the order Lactobacillales are involved in the fermentation of food, which has been used by humans for hundreds of years to preserve and refine food. The main function of Lactobacillales in food processing is to break down carbohydrates into lactic acid, which leads to the preservation of food by lowering the pH value. In addition, the bacteria perform functions that are typical of the genus or the species and are utilized on an industrial scale to specifically improve taste or other desirable properties. In contrast to the culturally empirical use of Lactobacillales, in industrial production, they are specifically added to the base product as so-called starter bacteria when processing of the basic product begins [6].

In contrast to most lactobacilli, *Leuconostoc* spp. is able to metabolize galactose via the Leloir pathway [6]. *Leuconostoc* spp. have an inducible citrate lyase, which is why these bacteria are often used in food production as starter bacteria for the metabolism of citrate, and at the end of the citrate decomposition process, CO_2_ is produced. In this way, *Leuconostoc* spp. contribute to the formation of eyes in Dutch-type cheese, whereas the eyes in Swiss-like cheese result from the metabolization of propionic acid to CO_2_ by propionibacteria [6]. The following *Leuconostoc* species are included in the list “Microorganisms with technological beneficial use”, which lists microorganisms used in the industrial production of food: *Leuconostoc carnosum*, *L. citreum*, *L. fallx*, *L. holzapfelli*, *L. inhae*, *L. lactis*, *L. mesenteroides*, *L. mesenteroides* subsp. *cremoris*, *L. mesenteroides* subsp. *dextranicum*, *L. mesenteroides* subsp. *mesenteroides*, and *L. palmae.* The bacteria are mainly used in the production of dairy products, but also in the processing of meat, fish, vegetables, coffee, and wine [7].

In addition to the search for the best possible conditions for the production of food, the use of *Leuconostoc* spp. as a pro- or prebiotic is currently being extensively researched. The results of the studies, particularly on laboratory animals, suggest that *Leuconostoc* could have a beneficial effect on human glucose and cholesterol metabolism, as well as mediating an anti-inflammatory effect, in addition to many other positive properties [8,9,10]. One of these effects could be a favorable effect on the composition of the intestinal microbiome. In in vitro studies, inhibition of the growth of human pathogenic bacteria mediated by various *Leuconostoc* species was observed [11,12,13,14]. Even the administration of heat-killed *Leuconostoc mesenteroides* improved the intestinal and renal dysfunction of mice suffering from chronic kidney failure [15]. While the results of the above analyses were obtained from specific strains of one *Leuconostoc* species, the interaction of several species belonging to the order Lactobacillales also appeared to impart favorable immunological properties. The cytokine patterns of T_Helper_ cells, isolated from volunteers who consumed kefir for a fortnight, suggested a TH2 to TH1 shift in the immune response. Furthermore, a reduced activation level of neutrophil granulocytes was observed indicating an anti-inflammatory activation level [16]. In addition, it was shown that the strain *Leuconostoc lactis* APC 3969 is able to produce the bacteriocin leucocyclicin Q, which in turn has an inhibitory effect on *Clostridium perfringens*. Therefore, the *Leuconostoc* strain could be used to reduce the risk of food contamination posed by *C. perfringens* [17]. Although the above findings are far from complete, the results presented suggest that *Leuconostoc* spp. and other Lactobacillales species have great probiotic potential.

*Leuconostoc* is not normally cultured from the stool of adults using classical cultural methods. In children, however, the bacteria have been successfully cultivated using selective media [18]. Since the 1980s, *Leuconostoc* spp. has been identified as the cause of mostly severe infections. To obtain an overview of the infections caused by different *Leuconostoc* species, a Pubmed search was performed using the search terms “*Leuconostoc* AND Infection”. This shows that the number of infections caused by *Leuconostoc* described in the last 50 years has remained relatively low. Table 1 summarizes the publications showing the infections caused by certain *Leuconostoc* species.

As shown in Table 1, most of the infections were caused by *Leuconostoc lactis* and *Leuconostoc mesenteroides*. In addition to the cases listed in the table, studies were found in which infections caused by *Leuconostoc* spp. were described, but no species identification was carried out [39,41,43,59,60,61,62,63,64,65,66,67,68,69,70,71,72,73,74,75,76,77,78,79,80,81,82,83,84,85]. The patients concerned often suffered from serious pre-existing conditions, i.e., lymphoma or leukemia [23,31,33,37,65,70], other cancers [26,33,45,57,67,69,86], cerebral bleeding [42,46,66], diabetes mellitus [38,55], other autoimmune diseases [27,29,47,76], organ failure or organ transplantation [25,62,71,77,78], and infections caused by other pathogens [30,36,73]. In contrast to earlier years [50,59,80], however, infections caused by *Leuconostoc* spp. in HIV patients are no longer reported today, presumably based on the available highly active antiretroviral therapy.

The analysis of several case series revealed the following risk factors for suffering from a *Leuconostoc* infection: intravascular catheters, antibiotic treatment, especially those with glycopeptide antibiotics, damage to the skin barrier, and neutropenia [20,68,70]. Lee et al. [28] analyzed a total of 20 infections caused by *Leuconostoc*. Fifteen infections had been caused by *L. lactis*, and a total of 19 were healthcare-associated. Eleven patients suffered from a tumor and had been hospitalized for more than 30 days. Bou et al. [33] analyzed the risk factors for contracting a *Leuconostoc* infection in a case-control study. The data of two outbreaks, one with 42 and one with six affected patients, had been analyzed. In the larger outbreak, eleven neonates and 31 adults were infected. Of the affected adults, nine had a solid tumor, one had leukemia, and five had lymphoma as their underlying disease. Previous infection and artificial feeding were identified as risk factors for infection with *L. mesenteroides* subsp. *mesenteroides*.

Various studies have shown that the administration of an ultimately effective antibiotic was successful after the previously placed catheter had been removed [40,41,79,87]. Huber et al. [73] had described that the clinical situation of an infected patient had even improved spontaneously after removal of the central venous catheter to such an extent that the administration of antibiotics was no longer necessary when the result of the species identification was finally available. In all, it can be seen that only adults who are very seriously ill anyway will suffer from a *Leuconostoc* infection.

In addition to severely ill adults, infections have also been observed in newborns and infants. Here, too, there is a common feature of serious life-threatening underlying diseases. The risk factors listed by the authors of the studies were essentially similar to those listed for adults. Practically, all of the children had had invasive catheters inserted, most of them required artificial feeding, and some of them had previously been treated with antibiotics [19,21,32,33,34,39,40,41,43,59,61,62,72,75,76,79,83,85,87,88,89].

In one of these cases, it had been possible to assign the origin of the infection. *Enterobacter sakazakii* and *L. mesenteroides* were detected as the blender for the rehydration of the dried baby food was investigated. These two species had previously been detected in blood cultures of a 6-month-old baby and were not found in the industrially produced dried food [34]. In all other cases, it ultimately remained unclear how the bacteria had found their way into the children’s bodies. There are only few reports in which *Leuconostoc* infections were observed in otherwise healthy individuals. This involves a 16-year-old girl who suffered from meningitis caused by *Leuconostoc*. The young lady survived the infection without neurological deficits [84]. The second case involved a 64-year-old man who fell ill with *Leuconostoc* that caused pleural empyema. He was professionally involved in the production and distribution of mixed pickles. As *Leuconostoc* is also used in the manufacture of this product, it could have been an infection in the course of his work. This patient also survived the infection [49].

For the present report, the infections caused by *Leuconostoc* spp. in our clinic were retrospectively analyzed. It was investigated whether *Leuconostoc* spp. was acquired in an outpatient setting or in hospital and which risk factors contributed to the development of nosocomial infections. Finally, the outcome of the *Leuconostoc* spp. infection was also determined. The case of a severely malnourished patient with two life-threatening *Leuconostoc* infections will be presented in detail.

## 2. Materials and Methods

Ingolstadt Hospital is located in the center of the Bavarian planning region 10, which has a population of approximately 500,000 and comprises the districts of Eichstätt, Neuburg-Schrobenhausen, and Pfaffenhofen, as well as the independent city of Ingolstadt [90]. The hospital is a second-level clinic and has 1073 beds. Around 3800 employees care for around 33,000 in- and day patients, as well as for 68,000 outpatients in 23 clinics, and institutes every year.

The hospital’s laboratory is accredited in accordance with DIN EN ISO/IEC 17011 [91]. Laboratory requests and their analysis results are managed with the software LabCentre l.i.c. Version 2025.05 (Mesalvo Mannheim GmbH, Manheim, Germany). The results of microbiological analyses are imported into the Hybase computer program V6.2025.01.R3 (epiNET GmbH, Bochum, Germany) for a systematic evaluation. The number of detections of certain microorganisms was obtained using the Hybase “Statistics” module. The first detection of a specific pathogen was queried per patient. If a patient with a blood culture was identified using the Hybase query, the laboratory program was used to analyze whether other microorganisms were also cultivated from the respective blood sample.

Microbiological tests are carried out in the laboratory of the hospital in accordance with the European Committee on Antimicrobial Susceptibility Testing (EUCAST) criteria. The BACT/ALERT VIRTUO system (BioMerieux, Nürtingen, Germany) was used to incubate blood culture bottles (Bact/Alert FA plus, Bact/Alert FN plus or Bact/Alert PF plus). Gram staining (Previ Color; BioMerieux) was performed on each blood culture with evidence of microbiological growth. From blood culture bottles exhibiting the growth of Gram-positive bacteria, a blood sample was cultured on Columbia agar with 5% blood (BioMerieux), as well as on chocolate agar (BioMerieux), and, in the case of the growth of anaerobe bacteria, additionally on Schädler agar (BioMerieux). When yeast fungi had been detected in the Gram preparation, a culture plate with Candida agar (BioMerieux) was also inoculated.

Punctates and biopsies were cultured in thioglycolate broth (BioMerieux) and subsequently grown on Columbia blood agar (BioMerieux). Parallel to this, punctate fluid was applied directly to Columbia blood agar using an eyelet.

Bacteria from various swabs were also cultivated on Columbia blood agar. Bacteria from rectal swabs were cultured on CHROMID^®^ CPS^®^ Elite agar/Columbia CNA agar + 5% sheep blood (Biomerieux). Gram-positive bacteria from urine were cultivated on Columbia blood agar plates containing 30 µg of nalidixic acid.

Species identification was performed using either mass spectrometry (Vitek MS; BioMerieux) or a Vitek 2 Compact and appropriate identification cards (Vitek GP or Vitek YS) from BioMerieux.

The antibiotic susceptibility of staphylococci and enterococci was tested using a Vitek 2 machine (BioMerieux) using the corresponding test kit from BioMerieux (AST-P654). For yeast, the test kit AST-YS08 (Biomerieux) was used. The minimum inhibitory concentration for penicillin, ampicillin, and vancomycin of *Leuconostoc* spp. was determined on Müller-Hinton agar with blood (BioRad, Neuried, Germany) using antibiotic-loaded strips (MIC-Teststreifen; BestiBion, Hürth, Germany).

The concentration of leukocytes in blood was analyzed with the DXH600 machine (Beckman Coulter, Krefeld, Germany), and the concentration of C-reactive protein (CRP), procalcitonin (PCT), and albumin was assessed with the Alinity machine (Abbott, Wiesbaden, Germany).

The observation period was January 2012 to March 2025. The data was analyzed retrospectively. Ingolstadt Hospital currently uses the hospital information system Cerner Soarian Clinicals version 4.7.100 (Cerner, Kansas City, MO, USA). The Soarian program contains an overview of every in- and outpatient stay of each patient. In addition, the corresponding diagnoses, all doctor’s letters, all examination results, and the notes documented by the nursing and medical staff are stored here, so that all the information required for the evaluation can be taken from the electronic patient file. The laboratory data of each patient is also imported into Soarian, so that laboratory data is also available there, albeit in a less convenient way than in the laboratory program.

Fisher’s exact test was used to calculate the significance level of the difference in the proportion of blood culture evidence and death rates of *Leuconostoc* spp.-colonized male and female patients. A Mann–Whitney-U test was applied to calculate the significance of other comparisons.

## 3. Results

**Figure 1:**
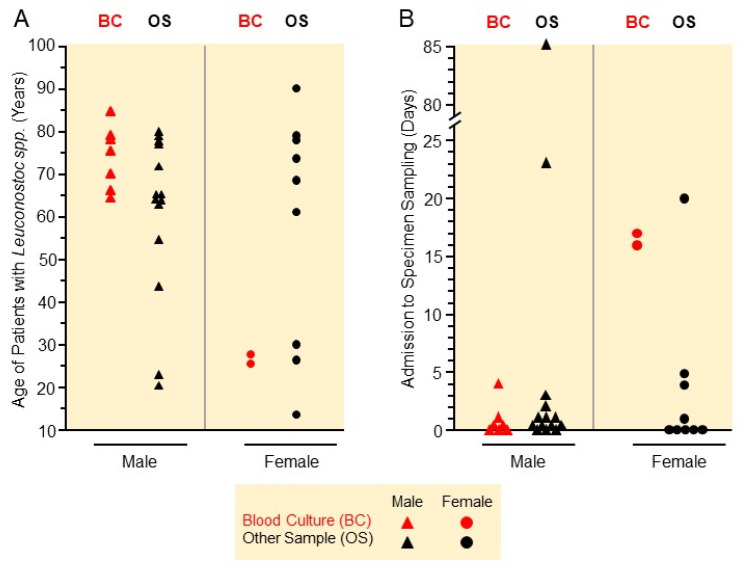
Male and female patients from whose materials *Leuconostoc* spp. were detected. (**A**) Age of patients. (**B**) Time interval between hospitalization and collection of patient samples from which *Leuconostoc* spp. were detected.

During the observation period from January 2012 to March 2025, *Leuconostoc* spp. were detected in clinical samples from a total of 32 patients (21 male, 11 female). As summarized in Table S1 in the Supplementary Materials, in a total of four patients (no. 10, 23, 24, 30), it was unlikely that the *Leuconostoc* spp. detection was due to infection, as the examined material was screening swabs from intact skin or mucous membranes, such as rectal, vaginal, and breast swabs. In the other 28 patients, it is at least possible that *Leuconostoc* spp. were involved in an infection. The median age of the affected patients was 65.2 years (average 60.5 years), whereby the median age of male (65.3 years) and female patients (67.8 years) did not differ significantly (Figure 1A). *Leuconostoc* spp. were isolated most frequently from samples taken on the day of admission (N = 14) or in the following five days (N = 10) or during the outpatient consultations of patients 10, 11, and 23 (Figure 1). The median time between hospitalization and collection of the sample from which the *Leuconostoc* spp. were obtained (0 days each) did not differ between male and female patients (Figure 1B).

While patients with blood culture evidence, in particular, suffered from very severe underlying diseases (Table 2), the severity of patients with evidence from other materials was quite heterogeneous (Table S1). A certain accumulation was seen in peripartal female patients (patients 8, 24, 28). Another accumulation was noticed in patients who had suffered acute limb injuries (patients 11, 16, 22) or who had recently undergone surgery for this reason and in whom the implant had become infected (patients 17, 28). In the case of the latter two, it is quite possible that the infection had been acquired during or after the operation that took place in a foreign hospital. However, as most patients already had been colonized or infected at the time of admission to the hospital or evidence of colonization was found within the first few days, nosocomial acquisition of the pathogen seems unlikely in these patients (Figure 1B). In the two female patients 8 and 9, *Leuconostoc* spp. were identified 17 and 18 days after hospitalization, and in the male patients 19 and 21, after 85 and 23 days. This constellation therefore makes it more likely that these patients acquired the bacteria nosocomially. Of these patients, only the two females survived, while the two males died. One of the two female patients (patient no. 8) had suffered a catheter-associated infection during childbirth. The second female patient (no. 9), on the other hand, had been severely malnourished and probably acquired the infection due to the immunosuppression associated with malnutrition. These two patients were also the only women in whom *Leuconostoc* spp. were detected in a blood culture. In contrast to these two women, the two male patients each had very serious underlying illnesses from which they would probably have died even without the *Leuconostoc* spp. infection (Table S1). The same applies to patients 1, 2, 3, 4, and 7 who died during their stay in which *Leuconostoc* spp. were detected in blood cultures (Table 2). In contrast to female patients, a total of seven male *Leuconostoc* spp.-infected patients died during their hospitalization (33%). However, this difference is not significant due to the small number of patients (*p* = 0.066).

*Leuconostoc* spp. were found in blood cultures from 7 of the 21 male (33.3%) and from 2 of the 11 female patients (18.2%) with proven colonization, suggesting that men have a higher risk of systemic infection when being colonized. In addition, male patients with *Leuconostoc* spp. in the blood culture were older (median age 75.5 years) than the two affected female patients (median age 26.8 years). As shown in Figure 1, the time between hospitalization and *Leuconostoc* spp. detection in blood culture was markedly longer in female (median duration 16.5 days) than in male patients (median 0 days).

As shown in Figure 1A, male patients with *Leuconostoc* spp. in the blood culture were older (median age 75.5 years) than males with *Leuconostoc* spp. in other body sites (median age 64.5 years). In contrast, female patients with *Leuconostoc* spp. in blood cultures with a median age of 26.8 years were younger than women exhibiting *Leuconostoc* spp. in other specimens (median age 68.8 years). However, these differences were also not significant due to the small number of patients.

As shown in Figure S2, there are no temporal clusters in the detection rates that would indicate the presence of an outbreak. *Leuconostoc* spp. were only isolated from two patients in each of three months, namely in June 2012, February 2013, and July 2019. In June 2012, patients no. 15 and 25 were treated in the same department and operated on 15 days apart. Since *Leuconostoc* spp. were isolated from the urine of patient no. 25, transmission in the operating theatre seems rather unlikely, although a doctor was present during both operations. In contrast, transmission between patients no. 23 and 31, who were treated in February 2013, appears to be extremely unlikely. While patient no. 23 had only presented to the urology outpatient clinic on 11 February for the collection of a rectal swab, from which *Leuconostoc* spp. was then detected, patient no. 31 was operated on by the pediatric surgeons on the fourth inpatient day. *Leuconostoc* spp. were isolated in both patients in July 2019 from samples taken on admission to our emergency department. A wound swab was taken from patient no. 16, who had suffered a serious traffic accident on 12 July, and a blood culture was taken from patient no. 3, who suffered from cancer, 11 days later.

From the blood cultures of the female patients, only *Leuconostoc* spp. were detected, while other bacteria were simultaneously isolated from the blood cultures of five of the seven male patients. *Leuconostoc* spp. accompanying bacteria were predominantly coagulase-negative staphylococci, but one patient additionally exhibited *Candida tropicalis* and *Enterococcus faecalis*.

The female patient, suffering from anorexia nervosa since the age of 14, had been an inpatient in psychiatric facilities and force-fed several times. Six weeks before the current hospital stay, she had already been hospitalized for the treatment of an erysipelas. At her current admission, the patient was 165 cm tall and weighed 23.5 kg resulting in a body-mass index (BMI) of 8.8 kg/m^2^. Her daily calorie intake was around 600–900 kcal, 300 kcal via a percutaneous endoscopic gastrostomy tube and 300 to 600 kcal via oral uptake. The reason for current hospitalization was dizziness and general malaise. On admission, there were no laboratory or clinical signs of infection (blood pressure 89/58, heart rate 73/min, pulse oximetry measured an oxygen saturation of 100%, body temperature 36.2 degrees Celsius). The initial examination revealed unremarkable findings, apart from a very pronounced cachexia. Echocardiography and abdominal sonography also provided unremarkable findings, which, however, could only be analyzed to a limited extent due to the physical constitution. A laboratory chemistry analysis confirmed previously known anemia and leukocytopenia. Initial enteral and parenteral nutrition were maintained and the patient additionally received parenteral nutrition (Olimel 500 kcal/d). Due to the normal phosphate, magnesium, and calcium concentrations, there was no indication of refeeding syndrome.

**Figure 2:**
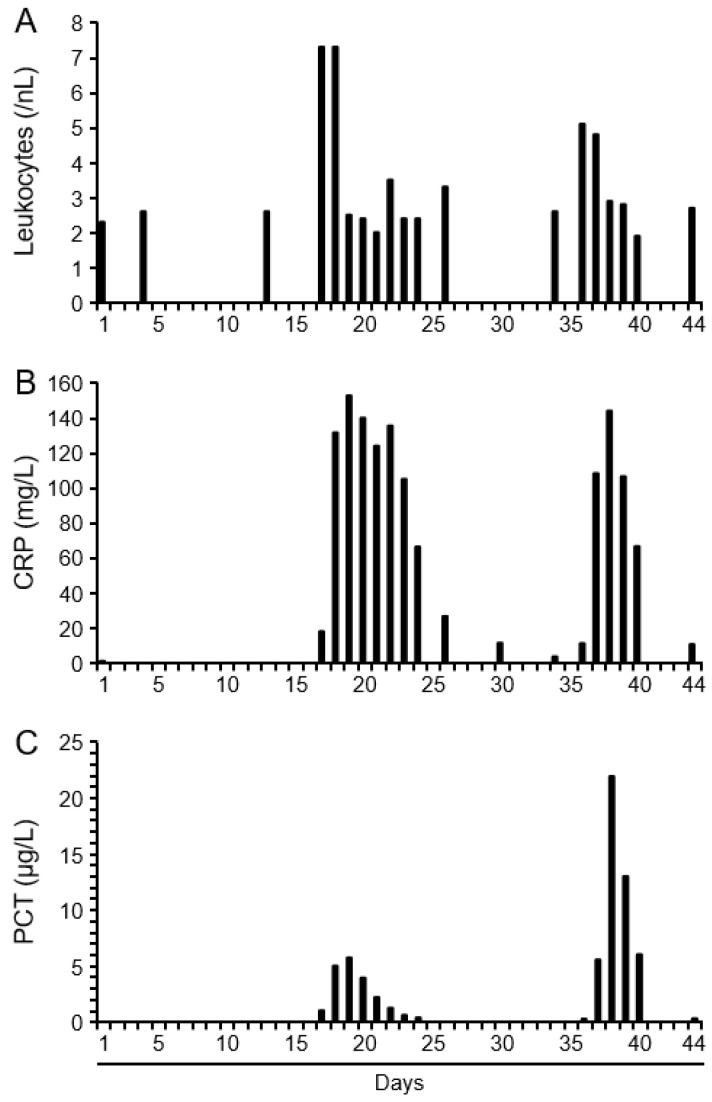
Serum concentration of leukocytes (**A**), C-reactive protein (CRP) (**B**), and procalcitonin (PCT) (**C**) in a female anorexia nervosa patient suffering from a *Leuconostoc* spp. infection.

On the 16th day after admission to the hospital, the patient developed a fever, which was the only symptom at that time. Blood pressure was similar to that at admission (86/52 mmHg), the heart rate was 117 beats per minute, and the temperature was 39.8 °C. Pulse oximetry showed an oxygen saturation of 97%. As shown in Figure 2, the concentration of leukocytes, CRP, and PCT increased markedly in the following days as a sign of a manifest systemic infection. However, despite the notable increase, the leukocyte concentration at 7.3/nL was still within the normal range. In contrast, the concentration of CRP at a maximum of 152.5 mg/L and that of PCT at 5.74 µg/L were well above the normal range (CRP: 3 mg/dL, PCT: 0.5 µg/L). X-ray examination of the chest revealed no evidence of an infiltrate, which is why a pulmonary infection seemed rather unlikely (Figure S3).

On the first day of the fever, two pairs (aerob/anaerob) of blood cultures had been taken, and *L. lactis* was detected in all four bottles (Table 3). Surprisingly, the growth of *L. mesenteroides* was obtained from a pair of blood culture bottles taken three days later. As EUCAST has not yet provided any reliable limit values for antibiotic susceptibility testing, it has not been possible to determine the resistance situation. As a rough guide, the minimum inhibitory concentrations of penicillin, ampicillin, and, for *L. lactis*, also vancomycin were measured. Due to the high concentrations required, both *Leuconostoc* species had to be considered as probably resistant to these antibiotics (Table 3).

Due to infection, the anorexic patient was transferred to the internal medical intensive care unit (ICU). Immediately after the first occurrence of fever, antibiotic treatment with piperacillin/tazobactam was started at a dosage adjusted to the patient’s body weight. After 6 days following the initial fever event, the patient was apyretic and was discharged from the intensive care unit. Piperacillin/tazobactam was given for a total of 14 days. After discontinuation of the antibiotics, the CRP and PCT concentration initially remained low (Figure 2).

However, seven days after the end of the antibiotic treatment, the patient developed a new fever. The other vital parameters were within the normal range for the patient. On the day of the flare-up, the CRP was elevated again and reached its maximum (143.8 mg/L) on the following day (Figure 2). At this time, the PCT also reached its highest value at 21.9 µg/L. Although this infection episode was a few days shorter than the first, this time, the PCT concentration was 4 times higher than during the first infection. Again, *L. mesenteroides* was detected in the blood cultures taken on the first day of fever.

The patient was again transferred to the ICU for treatment and monitoring. Antibiotic treatment with piperacillin/tazobactam was started again. This time, a CVC was inserted (for the first two days, then removed on the basis of the patient’s will). Under the antibiotic therapy, the infection parameters declined well and the patient was transferred back to the normal ward in a stable circulation/respiration situation. In the normal ward, the patient complained of increasing skin itching. An allergic reaction was suspected and the antibiotic therapy was switched to meropenem. Meropenem was administered for five days until the patient had recovered, accompanied by declining inflammatory markers. The patient was discharged home seven days after terminating the antibiotic therapy.

**Figure 3:**
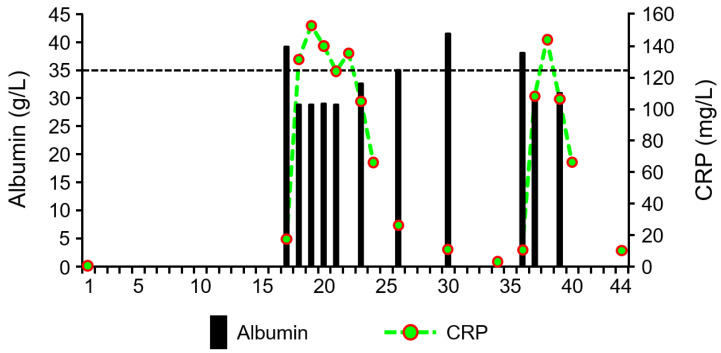
Albumin and C-reactive Protein (CRP) concentration in the serum of a female anorexia nervosa patient suffering from *Leuconostoc* spp. infection. Dotted line: lower limit of the normal value range of albumin.

During the period without infection, the albumin concentration was at the lower limit of the normal range (Figure 3). As the CRP concentration increased, the albumin concentration decreased even further to values below the normal range.

## 4. Discussion

In our monocentric study, the number of patients treated in our clinic who had *Leuconostoc* spp. in a clinical sample was retrospectively examined, and the risk factors for bloodstream infections caused by *Leuconostoc* spp. were analyzed. For the long observation period of more than 13 years, the number of patients colonized with *Leuconostoc* spp. was relatively low at N = 32, as was the number of blood culture detections, at N = 9. By comparison, *Escherichia coli* was isolated from blood cultures from 240 of our patients in 2024 alone.

As 21 (65.6%) of the 32 patients affected were male, men appear to be at a higher risk of colonization with *Leuconostoc* spp. than women. In addition, older men with severe underlying diseases have a high risk of developing a systemic infection with a fatal outcome as a result of colonization. This can be deduced from the finding that *Leuconostoc* spp. were isolated from blood cultures in half of the 14 affected male patients over the age of 60. However, we have only found one case report in Turkish, in which it is reported that *Leuconostoc* spp. can initially cause asymptomatic colonization before the development of a manifest infection [67].

It was recently reported, in a study conducted in India, that men suffer systemic infections significantly more frequently than women. Similar to our study, the patients affected had severe underlying diseases but were significantly younger with a median age of 34.5 years. This could explain why the mortality of patients with blood culture evidence in the Indian study was only half as high as in the present study. [20]. However, the Indian study did not report whether the infections were acquired nosocomially or on an outpatient basis. However, since the authors report that eight of the 14 affected patients were hospitalized for more than 30 days, the authors obviously assume that the infections were acquired in the hospital. A similar constellation was found by Lee et al. [28]. In their analysis, 19 of 20 infections were hospital-acquired, with 11 patients having been in the hospital for more than 30 days before the bacteremia was detected. In the study by Bou et al. [33], pulsed-field gel electrophoresis analyses were carried out, in addition to microbiological diagnostics. As far as can be seen from that manuscript, four different band patterns were obtained, which indicates a heterogeneous population. However, as one band pattern prevailed during each of the two outbreaks, nosocomially acquired infections can be assumed in this case.

The original aim of the authors of the present study was also to investigate risk factors for *Leuconostoc* spp. infection in patients at our clinic. However, the finding that *Leuconostoc* spp. were isolated from samples taken on the first or during the first days of hospitalization suggests that the bacteria were acquired almost exclusively on an outpatient basis. Therefore, it was of course no longer meaningful to analyze the risk factors of a nosocomially acquired bloodstream infection in this study. On the other hand, the literature review presented in the introduction shows that almost all patients who had suffered from a systemic *Leuconostoc* spp. infection had suffered from very severe underlying diseases, most of which were fatal even without an additional infection. Almost all patients with such underlying conditions have intravascular catheters, receive antibiotic treatments, and suffer from skin barrier destruction and/or neutropenia. It is therefore unsurprising that these factors have been identified as a risk for systemic *Leuconostoc* spp. infections [20,68,70]. However, our results show that the presence of a nosocomial infection must first be clarified.

Two bloodstream infections had occurred in young female patients without limiting pre-existing conditions. As will be discussed in detail below, the infection in patient no. 9 probably occurred in the context of her immunologically desolate general condition. Patient no. 8 had suffered from thrombophlebitis prior to the blood culture detection. It is therefore very likely that the systemic infection had developed on the basis of a catheter infection. In various case descriptions, the removal of indwelling catheters has contributed to the successful treatment of *Leuconostoc* spp. infections [40,41,73,79,87]. Some authors have even described that the removal of the colonized catheter led to the healing of the infection even without additional antibiotics [41,71]. Therefore, it seems plausible that catheters represent a portal of entry for nosocomial bloodstream infections and should be removed in the event of *Leuconostoc* spp. blood culture evidence. In a study on blood donors, Celere et al. [92] described that *L. mesenteroides* and *Staphylococcus hominis* can evade skin disinfection. In this respect, the occurrence of catheter-associated infections does not automatically mean that the usual hygiene measures were disregarded before the occurrence of device-associated infections.

A reduced sensitivity to skin disinfectants, as has also been demonstrated for other microorganisms [93,94,95], could have contributed to the fact that in the present study, as in earlier descriptions [20,22,25,34,82,96], other microorganism species were detected in blood cultures. As the multiple detection was based on blood cultures taken right at the beginning of the hospitalization, the damaged barrier is likely to have played a major role in the detection of multiple species.

Intuitively, it should be assumed that patients suffering from anorexia nervosa are particularly susceptible to infections. In fact, however, the opposite seems to be true. Although it is currently assumed that infections may trigger the development of eating disorders [97,98], patients suffering from anorexia nervosa actually do not suffer more frequently from viral and bacterial infections, although a number of immunosuppressive effects have been demonstrated for these patients [99]. However, there appear to be two exceptions to this. The first concerns infections with mycobacteria, regardless of whether they are *Mycobacterium tuberculosis* complex or atypical mycobacteria [100,101,102]. The second exception is that an extremely low body weight can lead to bacterial infections, regardless of whether the causative bacterial species exhibit certain pathogenicity factors or not. If the BMI falls below a threshold of about 12 kg/m^2^, bacterial infections can become life threatening even from otherwise less pathogenic bacteria [103]. Therefore, the cause of the infections that occurred in patient 9 must be attributed primarily to the patient’s destructive nutritional condition and to her venous access and not to the high pathogenicity of the two bacterial species. Patient 9 had already been fitted with a PIVC before the infection occurred. Hirata et al. [104] had observed a high rate of catheter-associated infections in patients with severe AN (median BMI 12.2 kg/m^2^), and those patients who had to be physically restrained were at the highest risk. Consistent with this, our patient exhibited a great urge to exercise during the supplementation period and was also busy running around her bed even in the ICU. In our view, it would therefore not be appropriate to remove *Leuconostoc* species from the list of “Microorganisms with technological beneficial use” [6].

Despite the critical nutritional status, the albumin concentration of patient 9 was still in the lower normal range as long as there was no infection. This constellation, which may seem surprising at first, has already been observed in patients suffering from anorexia nervosa [105]. During the infection episodes, however, the albumin concentration dropped to values below the normal range. During the episodes, the concentration of CRP increased considerably. However, the increase was more moderate than would have been expected from the level of the PCT concentration. It has recently been described that the level of CRP concentrations in infections of anorexia nervosa patients is limited [106]. Both proteins are produced in the liver. Obviously, the amount of amino acids available in such malnourished patients is not sufficient to maintain both a normal albumin concentration and adequate CRP production.

The authors are currently not aware of any study in which a *Leuconostoc* infection associated with anorexia nervosa has been described. However, there are few reports of infections caused by *Leuconostoc* spp. in malnourished infants [89]. The finding that two *Leuconostoc* infections occurred approximately 30 days apart is also described here for the first time. It is also remarkable that the first episode of infection was essentially caused by *L. lactis* and the second by *L. mesenteroides*, a constellation previously unknown to us.

EUCAST has not yet issued any recommendations for antibiotic testing and the interpretation of test results for *Leuconostoc* spp. There are also various reports of patients dying from infections caused by *Leuconostoc* spp. even though the antibiotics administered should have been effective [28,48]. The successful treatment of patients no. 5, 6, 8, and 9, who had suffered from a systemic infection including blood culture evidence, was therefore pleasing.

Our study has limitations. Several authors have stated that sequencing of the 16S rRNA-coding DNA sequence is required for unambiguous species identification [25,28,107]. Unfortunately, our laboratory does not have the necessary technical facilities for DNA sequencing. On the other hand, two different *Leuconostoc* species were isolated from blood cultures from patient 9. *L. lactis* required substantially shorter incubation times until growth was detected than *L. mesenteroides*. However, the incubation times among the isolates of the same species were quite similar. Similarly, the *L. lactis* isolates showed considerably higher MICs in the antibiotic susceptibility test than the *L. mesenteroides* isolates, although, again, the isolates of the same species showed identical MICs to each other. These findings indicate that at least the identification of two different *Leuconostoc species* was correct for this patient.

Another weakness is that the present analysis is only descriptive and not a systematic analysis. However, a prospective study would require a disproportionately long observation period, so this form of analysis was ruled out anyway. A further shortcoming is that the differences found between men and women were not statistically significant. This is due to the small number of cases. Therefore, further studies with larger numbers of cases are required in order to verify the trends presented here.

Overall, the results of our study show that infections caused by *Leuconostoc* spp. are relatively rare. Systemic infections almost exclusively affect older males with serious, usually fatal underlying diseases whereby colonization and the development of an infection in these patients occurs in the outpatient sector. The mechanisms involved should be the subject of further investigation.

## 5. Conclusions

Colonization with *Leuconostoc* spp. occurs predominantly in the outpatient sector. Systemic infections, which are often fatal, mainly affect men over the age of 60 with serious underlying illnesses. However, women can suffer systemic infections in the context of nosocomial events, whereby catheter infections are likely to be the cause.

## Figures and Tables

**Table 1:** Literature overview of different *Leuconostoc* species and the infections they caused. The studies listed were retrieved based on a PubMed search using the search terms “*Leuconostoc* AND Infection”.

	*L. lactis*	*L. mesenteroides*	*L. pseudomesenteroides*	*L. citreum*	Other Species
Systemic infection	Azghar et al. [19] Tripathy et al. [20] Gagliardo et al. [21] Hosoya et al. [22] Matsuda et al. [23] Yang et al. [10,24] Deng et al. [25] Patel et al. [26] Shin et al. [27] Lee et al. [28]	Gordon et al. [29] Menegueti et al. [30] Ino et al. [31] Florescu et al. [32] Bou et al. [33] Noriega et al. [34] Immel et al. [35] Valencia et al. [36]	Simoiu et al. [37] Ghobrial et al. [38] Carapetis et al. [39]	Modaweb et al. [40] Bernaldo de Quirós [41] Taşkapılıoğlu et al. [42]	*L. dextrani-cum*: Buu-Hoi [43] *L. cremoris*: Jiménez-Mejías et al. [44]
CNS infection	Omori et al. [45] Deye et al. [46]	Albanese et al. [47] Friedland et al. [48] Valencia et al. [36]		Taşkapılıoğlu et al. [42]	
Endocarditis		Valencia et al. [36]			
Lung infection		Usta-Atmaca et al. [49]		Giacometti et al. [50]	
Eye infection		Damasceno et al. [51]			
Periprosthetic knee infection		Franco-Cendejas et al. [52]			
Urinary infection		Taneja et al. [53]	Cappelli et al. [54]		
Liver abscesses	Vagiakou-Voudris et al. [55]				
Hemophagocytic lymph-histiocytosis		Lin et al. [56]			
Osteomyelitis		Mohta et al. [57] Zaoui et al. [58]			

**Table 2:** Patients exhibiting *Leuconostoc* spp. in blood cultures at Ingolstadt Hospital between January 2012 and March 2025. F = Female, M = Male, D = Death, S = Survival.

Pat. No.	Age	Sex	Microorganisms in Blood Culture	Underlying Disease	Outcome
1	70	M	*Leuconostoc* spp.	Diffuse large B-cell lymphoma	D
2	78	M	*L. mesenteroides*	Intracerebral hemorrhage	D
3	75	M	*L. mesenteroides*	Malignant melanoma	D
4	84	M	*L. mesenteroides*, *S. epidermidis*, *S. auricularis*	Parkinson’s disease, Obstructive ileus	D
5	65	M	*L. mesenteroides*, *S. epidermidis*	Bronchial carcinoma	S
6	64	M	*L. mesenteroides*, *S. epidermidis*	Cardiac arrest, Coronary artery disease	S
7	76	M	*Leuconostoc* spp., *E. faecalis*, *C. tropicalis*, *S. haemolyticus*	Carotid artery occlusion	D
8	27	F	*Leuconostoc* spp.	Peripartal thrombophlebitis	S
9	26	F	*L. lactis*, *L. mesenteroides*	Anorexia nervosa	S

**Table 3:** Characteristics of *Leuconostoc* spp. isolated from blood cultures of a female anorexia nervosa patient during two infection episodes. MIC: minimal inhibitory concentration. HH:MM = hours:minutes.

Date	Blood Culture No.	Blood Culture Mode	Species	Time to Positivity (HH:MM)	MIC (µg/mL)
Episode 1					
28.01.	1	Aerobe	*L. lactis*	16:32	Penicillin 0.75 Ampicillin 1.0 Vancomycin > 256
“	1	Anaerobe	“	11:02	
28.01.	2	Aerobe	*L. lactis*	17:32	
“	2	Anaerobe	“	10:23	
31.01.	3	Aerobe	*L. mesenteroides*	23:46	Penicillin 0.38 Ampicillin 0.75
“	3	Anaerobe	“	25:55	
Episode 2					
17.02.	4	Aerobe	*L. mesenteroides*	21:15	Penicillin 0.38 Ampicillin 0.75
“	4	Anaerobe	“	23:45	
17.02.	5	Aerobe	*L. mesenteroides*	24:25	
“	5	Anaerobe	“	22:35	

## Data Availability

The raw data supporting the conclusions of this article will be made available by the authors on request.

## Supplementary Materials

The following supporting information can be downloaded at: https://www.mdpi.com/article/10.3390/microorganisms13071626/s1, Figure S1: Systematic classification of the genus Leuconostoc according to the classification of Euzéby & Parte [3]; Figure S2: Time course of the Leuconostoc ssp. isolations; Figure S3: Chest X-ray of patient 9; Table S1: Patients with *Leuconostoc* spp. detection from patient’s specimen between January 2012 and March 2025. Shown are the date of hospital admission and discharge as well as the period (days) from admission to collection of the specimen and the underlying diseases.

## Abbreviations

The following abbreviations are used in this manuscript:

spp.	Species
EUCAST	European Committee on Antimicrobial Susceptibility Testing
CRP	C-reactive protein
PCT	Procalcitonin
CVC	Central venous catheter
PIVC	Peripheral intravenous catheter
PEG	percutaneous endoscopic gastrostomy
BMI	Body mass index
MIC	Minimum inhibitory concentration
ICU	Intensive care unit
BC	Blood culture
OS	Other sample
PSA	Prostate-specific antigen
NSTEMI	Non-ST-elevation myocardial infarction
