# Supplementary material for: Both, Limited and Often Fatal Systemic Infections Caused by Leuconostoc spp. in Older, Previously Ill Men Are Usually Acquired in the Outpatient Setting
Source: Microorganisms. 2025 Jul 10;13(7):1626. doi: 10.3390/microorganisms13071626 (PMC12298493; doi:10.3390/microorganisms13071626)
Supplement: Supplementary file 1 [file microorganisms-13-01626-s001.zip › Table S1.pdf]

Table S1: Patients with *Leuconostoc spp.* detection from patient´s specimen between Jan 2012 and Mar 2025. Shown are the date of hospital admission and discharge as well as the period (days) from admission to collection of the specimen and the underlying diseases.

Patient No.	Sex	Age	Admission	Sampling	Specimen Sampling	Dis-charge	Specimen	Underlying Disease
1	M	70.0	21 Apr	21 Apr	0	22 Apr	Blood culture	Diffuse large B-cell lymphoma
2	M	78.7	02 Aug	06 Aug	4	09 Aug	Blood culture	Intracerebral hemorrhage
3	M	75.8	23 Jul	23 Jul	0	18 Aug	Blood culture	Malignant melanoma
4	M	84.3	01 May	01 May	0	05 May	Blood culture	Parkinson's disease, Obstructive ileus
5	M	65.3	21 Jan	21 Jan	0	11 Feb	Blood culture	Bronchial carcinoma
6	M	64.2	01 Sep	01 Sep	0	16 Sep	Blood culture	Cardiac arrest, Coronary artery disease
7	M	77.9	23 Dec	25 Dec	2	05 Jan	Blood culture	Carotid artery occlusion
8	W	27.8	07 Jan	24 Jan	17	03 Feb	Blood culture	Thrombophlebitis
9	W	25.8	12 Jan	28 Jan	16	06 Mar	Blood culture	Anorexia nervosa
10	M	77.2	09 Oct	09 Oct	0	09 Oct	Rectal swab	Suspicious palpation findings of the prostate and increased PSA concentration
11	M	22.6	02 Jun	02 Jun	0	02 Jun	Wound biopsy	Infected knee wound after football match
12	M	78.9	03 Jan	04 Jan	1	21 Jan	Wound swab	Peripheral occlusive disease, wet necrosis toe DII right
13	M	77.4	06 May	06 May	0	08 Jun	Intraoperative swab	Ulcus ventriculi
14	M	54.8	01 Aug	01 Aug	0	08 Aug	Intraoperative swab	Oesophageal carcinoma

Table S1: continued

Patient No.	Sex	Age	Admission	Sampling	Specimen Sampling	Dis-charge	Specimen	Underlying Disease
15	M	20.6	10 Jun	11 Jun	1	13 Jun	Intraoperative swab	Pilonidal sinus
16	M	63.9	12 Jul	12 Jul	0	19 Jul	Wound biopsy	Accident with scooter, elbow and forearm trauma
17	M	64.8	05 Nov	06 Nov	1	16 Nov	Wound biopsy	Arthrodesis of the right upper ankle joint with calming down infection
18	M	64.2	12 Dec	15 Dec	3	20 Dec	Punctate	Acute outpatient infection after previous prostate vesiculectomy
19	M	43.5	06 Jan	01 Apr	85	15 Apr	Abscess punctate	Necrotising pancreatitis
20	M	63.8	26 May	27 May	1	11 Jun	Ascites punctate	Non-small cell lung cancer, death 16.07.2024
21	M	79.8	09 Aug	01 Sep	23	10 Sep	Punctate of drainage fluid	Acute cholecystitis, NSTEMI
22	M	65.0	27 Jul	27 Jul	0	27 Sep	Wound biopsy	Extensive bimalleolar fracture of the upper jumper's joint on the right, decollment of the knee and thigh on the left
23	M	71.7	11 Feb	11 Feb	0	11 Feb	Rectal swab	Prostate carcinoma
24	W	30.2	22 Oct	27 Oct	5	27 Oct	Vaginal swab	Pre-term labor
25	W	61.3	02 Jun	22 Jun	20	22 Aug	Urine sample	Rectal perforation with fascial necrosis
26	W	79.1	28 Feb	01 Mar	1	23 mar	Intraoperative swab	Incarcerated incisional hernia with abscess and abdominal wall necrosis following previous surgery for breast cancer
27	W	78.5	01 May	01 May	0	02 May	Urine sample	Decompensated heart failure, previous sepsis
28	W	73.9	11 Mar	11 Mar	0	13 Apr	Wound biopsy	Removal of a plate osteosynthesis due to a low-grade infection
29	W	68.8	31 May	31 May	0	04 Jun	Wound swab	Grade 4 peripheral arterial occlusive disease
30	W	26.6	09 Jun	09 Jun	0	09 Jun	Breast swab	Suspicion of pre-eclampsia
31	W	13.7	21 Feb	25 Feb	4	03 Mar	Wound swab	Appendicitis
32	W	90.2	21 Jan	21 Jan	0	27 Jan	Wound swab	Diabetic ulcer, rectal carcinoma

PSA: Prostate specific antigen; NSTEMI: Non-ST-Elevation Myocardial Infarction
