# Supplementary figures and images for: Both, Limited and Often Fatal Systemic Infections Caused by Leuconostoc spp. in Older, Previously Ill Men Are Usually Acquired in the Outpatient Setting
Source: Microorganisms. 2025 Jul 10;13(7):1626. doi: 10.3390/microorganisms13071626 (PMC12298493; doi:10.3390/microorganisms13071626)

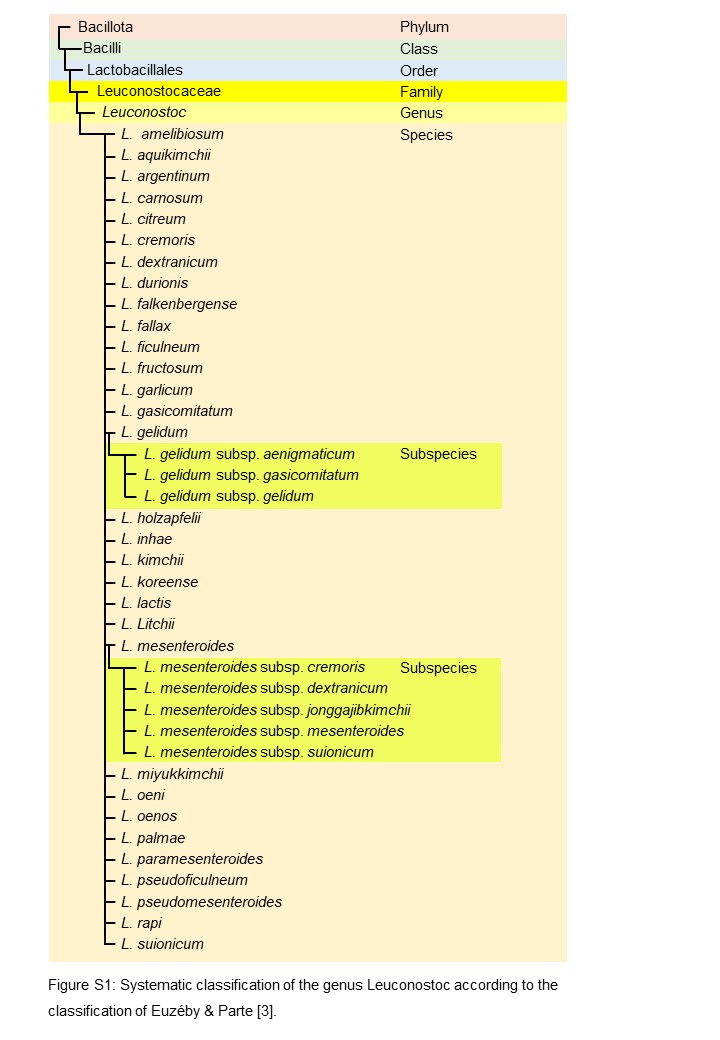

Supplement: Supplementary file 1 [file microorganisms-13-01626-s001.zip › Figure S1.jpg]

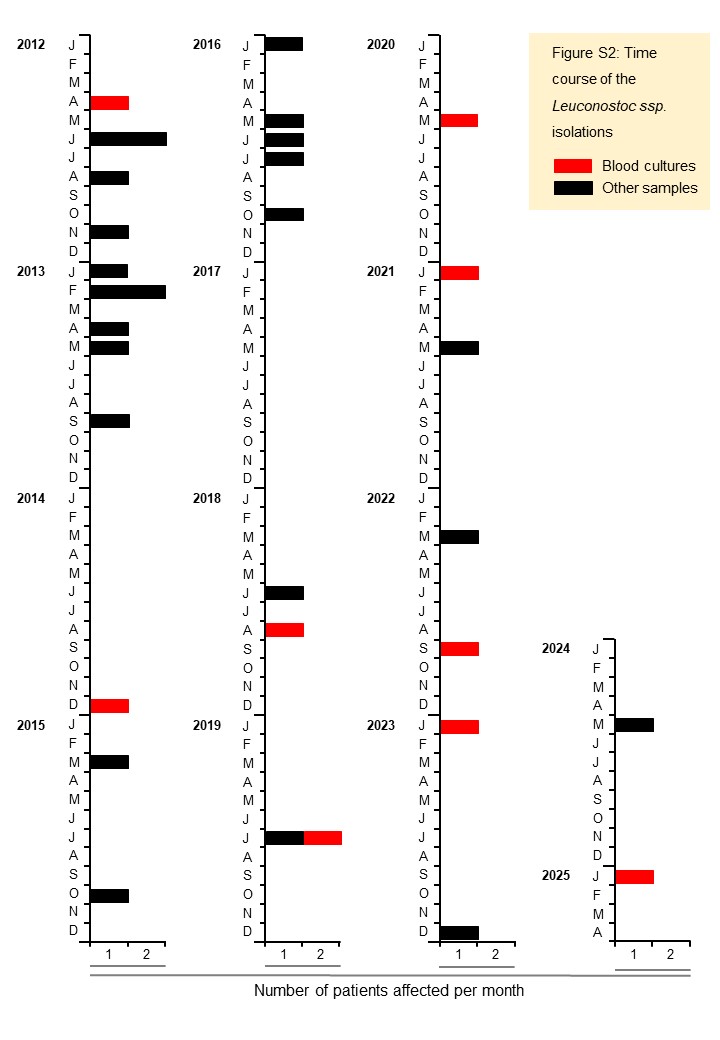

Supplement: Supplementary file 1 [file microorganisms-13-01626-s001.zip › Figure S2.jpg]

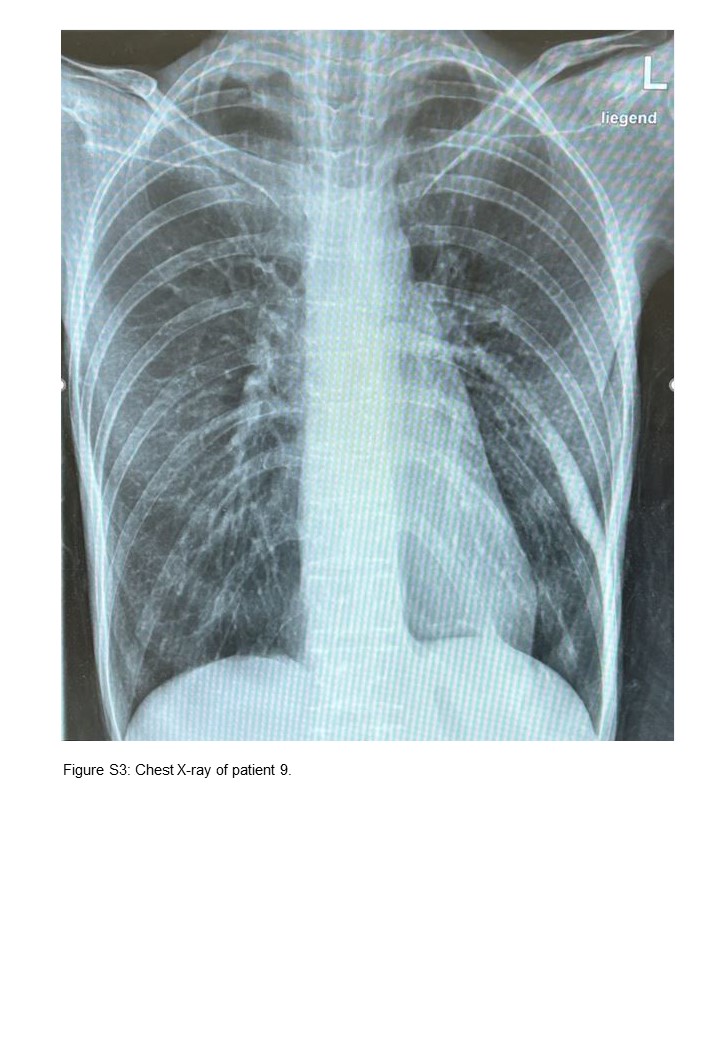

Supplement: Supplementary file 1 [file microorganisms-13-01626-s001.zip › Figure S3.jpg]
